# Development of a New Genus-Specific Quantitative Real-Time PCR Assay for the Diagnosis of Scrub Typhus in South America

**DOI:** 10.3389/fmed.2022.831045

**Published:** 2022-04-27

**Authors:** Ju Jiang, Constanza Martínez-Valdebenito, Thomas Weitzel, Christina M. Farris, Gerardo Acosta-Jamett, Katia Abarca, Allen L. Richards

**Affiliations:** ^1^Naval Medical Research Center, Silver Spring, MD, United States; ^2^The Henry M. Jackson Foundation for the Advancement of Military Medicine, Inc., Bethesda, MD, United States; ^3^Departamento de Enfermedades Infecciosas e Inmunología Pediátrica, Escuela de Medicina, Pontificia Universidad Católica de Chile, Santiago, Chile; ^4^Laboratorio Clínico, Clínica Alemana de Santiago, Facultad de Medicina Clínica Alemana, Universidad del Desarrollo, Santiago, Chile; ^5^Instituto de Ciencias e Innovación en Medicina (ICIM), Facultad de Medicina Clínica Alemana, Universidad del Desarrollo, Santiago, Chile; ^6^Instituto de Medicina Preventiva Veterinaria, Facultad de Ciencias Veterinarias, Universidad Austral de Chile, Valdivia, Chile; ^7^Programa de Investigación Aplicada en Fauna Silvestre, Facultad de Ciencias Veterinarias, Universidad Austral de Chile, Valdivia, Chile; ^8^Department of Preventive Medicine and Biostatistics, Uniformed Services University of the Health Sciences, Bethesda, MD, United States

**Keywords:** scrub typhus, *Orientia*, *Candidatus* Orientia chiloensis, molecular diagnostic techniques, quantitative real-time PCR (qPCR), Orien16S, South America, Chile

## Abstract

Scrub typhus is a potentially severe rickettsiosis, caused by *Orientia tsutsugamushi* in the Asia-Pacific region. Recently, however, two distinct pathogens, “*Candidatus* Orientia chuto” and “*Candidatus* Orientia chiloensis”, have been discovered in the Middle East and South America, respectively. Since the novel pathogens differ significantly from *O. tsutsugamushi*, many established diagnostic methods are unreliable. This work describes the development and validation of a new quantitative real-time PCR (qPCR) assay (Orien16S) for the detection of all known *Orientia* species. Based on a 94 bp sequence of the 16S rRNA gene (*rrs*), Orien16S recognized DNA samples from *O. tsutsugamushi* (*n* = 41), *Ca.* O. chiloensis (*n* = 5), and *Ca.* O. chuto (*n* = 1), but was negative for DNA preparations from closely related rickettsiae and other members of the order Rickettsiales (*n* = 22) as well as unrelated bacterial species (*n* = 11). After its implementation in Chile, the assay was verified, correctly identifying all tested eschar and buffy coat samples (*n* = 28) of clinical suspected cases. Furthermore, Orien16S detected *Orientia* DNA in trombiculid mites collected in endemic regions in southern Chile. The presented novel qPCR assay provides a useful tool for detecting *Orientia* and diagnosing scrub typhus from all geographical regions.

## Introduction

*Orientia*, one of the two genera of the family Rickettsiaceae in the order Rickettsiales, was previously considered to contain only a single species, *Orientia tsutsugamushi*, and to be confined to an area designated as the tsutsugamushi triangle, which includes areas in Asia, Australia, and islands of the Indian and Pacific Oceans (1, 2). Until 2010, this obligate intracellular pathogen was considered the exclusive cause of greater than 1 million annual cases of scrub typhus, a severe rickettsiosis with significant mortality (1). However, several autochthonous cases of scrub typhus have recently been reported on Chiloé Island and in other parts of southern Chile (3–5). Based on genetic analyses of 18 DNA preparations of clinical samples from scrub typhus patients, the pathogen causing scrub typhus in South America was found to be genotypically distinct to *O. tsutsugamushi* and *Candidatus* Orientia chuto (6), therefore currently designated as “*Candidatus* Orientia chiloensis” (7). *Ca.* O. chuto was originally described from samples of a patient returning from the United Arab Emirates (6). Sequences related to this organism were detected in mite samples collected from a village in Kenya (8), where humans were also seroreactive to *O. tsutsugamushi*, suggesting a wider, possibly global risk of scrub typhus caused by different species of *Orientia* (2).

Molecular analyses are the key diagnostic tools for the identification of new species of the order Rickettsiales and other intracellular bacteria (9). For the initial detection of scrub typhus cases in Chile, established molecular markers for *O. tsutsugamushi*, targeting sequences of the genes for 16S rRNA (*rrs*), 56-kDa type-specific antigen (*tsa*), and 47-kDa high temperature requirement A antigen (*htrA*), were used (3, 4). However, results were inconsistent, and several probable cases showed negative results using these classical targets (authors’ unpublished observations). Therefore, in 2017, the Naval Medical Research Center and the Chilean Rickettsia and Zoonosis Research Group designed a novel quantitative real-time PCR (qPCR) assay named Orien16S targeting a fragment of the *Orientia* genus-specific *rrs* sequence. Since then, the new assay has been evaluated and successfully applied to diagnose further human scrub typhus cases in Chile (5, 10). Furthermore, the test was used to detect *Orientia* DNA in trombiculid mites collected from Chiloé Island (11). Herein we present the technical details of the development of this assay, the validation process using a broad DNA panel of *Orientia*, *Rickettsia*, and other bacteria species, the comparison to an established qPCR assay for *O. tsutsugamushi* (Otsu47) (12), and its real-world performance in research laboratories in Chile with clinical samples and mite specimens.

## Materials and Methods

### DNA Samples

The panel used to determine the analytical performance of the novel qPCR assay (Orien16S) included DNA samples from: (1) *Orientia* species [*O. tsutsugamushi* (*n* = 41), *Ca*. O. chiloensis (*n* = 5), and *Ca.* O. chuto (*n* = 1)]; (2) *Rickettsia* species (*n* = 18); (3) Anaplasmataceae species (*n* = 4), (*Anaplasma phagocytophilum*, *Ehrlichia chaffeensis*, *Neorickettsia risticii*, and *Neorickettsia sennetsu*); (4) unrelated bacterial species (*n* = 11); and (5) human DNA (*n* = 1) (Roche Applied Sciences, Indianapolis, IN, United States) and normal mouse DNA (*n* = 4) (Table 1). DNA samples of *Ca*. O. chiloensis were extracted from a serum sample of the first scrub typhus patient (3), and eschar material (biopsy or swab) of four further cases from Chiloé Island and continental Chile diagnosed in 2015 and 2016 (5). The presence of the *Orientia* DNA in Chilean patient samples was confirmed by PCR and sequencing of the *rrs* and *htrA* genes. BLAST*™* searches in GenBank^1^ showed the closest matches to the sequences were *Orientia* species and subsequent phylogenetic analyses grouped them as *Ca*. O. chiloensis (7). The single *Ca.* O. chuto DNA sample was extracted from cell culture derived from a blood specimen of the unique scrub typhus case from the Arabian Peninsula (6); sources of the remaining DNA samples of *O. tsutsugamushi* and other microorganisms have been described previously (12–14).

**TABLE 1:** DNA preparations from bacterial strains and other sources included in the validation of the novel qPCR assay, Orien16S.

*Orientia tsutsugamushi* strains (origin)		New *Orientia* agents (origin)	Other Rickettsiales agents	Other bacteria and controls
			
*n* = 41		*n* = 6	*n* = 22	*n* = 16
Karp (New Guinea)	Woods (Australia)	*Ca*. O. chuto (United Arab Emirates)	*Rickettsia africae* ESF-5	*Salmonella enterica*
Kato (Japan)	Sido (Australia)	*Ca.* O. chiloensis Pt1 (Chile)	*Rickettsia akari* 29	*Proteus mirabilis*
Gilliam (Burma)	BSR178 (New Zealand)	*Ca.* O. chiloensis Pt2 (Chile)	*Rickettsia australis* PHS	*Escherichia coli*
AFC-3 (Thailand)	Buie (New Guinea)	*Ca.* O. chiloensis Pt3 (Chile)	*Rickettsia amblyommatis* 85-1084	*Corynebacterium* sp.
AFC-30 (Thailand)	Calcutta (India)	*Ca.* O. chiloensis Pt4 (Chile)	*Rickettsia bellii* G2D	*Legionella pneumophila*
AFPL-12 (Thailand)	Ikeda (Japan)	*Ca*. O. chiloensis Pt5 (Chile)	*Rickettsia canadensis* CA410	*Bartonella vinsonii*
TA-678 (Thailand)	Kawasaki (South Korea)		*Rickettsia conorii* ITT	*Bartonella quintana*
TA-686 (Thailand)	18-032111 (Pakistan)		*Rickettsia felis* URRWXCal2	*Francisella persica*
TA-763 (Thailand)	18-032460 (Malaysia)		*Rickettsia honei* TT-118	*Staphylococcus aureus*
TH-1812 (Thailand)	18-030642 (China)		*Rickettsia japonica* NK	*Borrelia burgdorferi*
TH-1814 (Thailand)	MAK-110 (China-Taiwan)		*Rickettsia montanensis* OSU 85-930	*Coxiella burnetii*
TH-1817 (Thailand)	MAK-119 (China-Taiwan)		*Rickettsia parkeri* C	Human DNA
CRF136 (Thailand)	MAK-243 (China-Taiwan)		*Rickettsia prowazekii* Breinl	Mouse DNA 1
FPW1038 (Thailand)	TM1073 (Laos)		*Rickettsia rhipicephali*	Mouse DNA 2
FPW2016 (Thailand)	TM1324 (Laos)		*Rickettsia rickettsii* R	Mouse DNA 3
UT76 (Thailand)	Faulkner (Vietnam)		*Rickettsia sibirica* 246	Mouse DNA 4
UT221 (Thailand)	Hicks (Vietnam)		*Rickettsia slovaka* Arm25	
UT661 (Thailand)	Middleton (Vietnam)		*Rickettsia typhi* Wilmington	
Brown (Australia)	Volner (Philippines)		*Anaplasma phagocytophilum*	
Citrano (Australia)			*Ehrlichia chaffeensis*	
Domrow (Australia)			*Neorickettsia risticii*	
Garton (Australia)			*Neorickettsia sennetsu*	

A plasmid (pOrien6) with the target *rrs* sequence for Orien16S and *htrA* sequence for Otsu47 assays was designed using gene fragment synthesis and cloning (Eurofins Genomics, Louisville, KY, United States). We used pOrien6 not only as a positive control, but also to develop a standard curve to quantitate genome equivalents in samples and to define the limit of detection (LOD) of the Orien16S assay. The dried plasmid was resuspended in 1× TE buffer and the concentration of the plasmid was calculated. Tenfold serial dilutions were made ranging from 5 × 10^7^ to 5 × 10^2^ copies/μl, followed by half log serial dilutions down to 5 × 10^–2^ copies/μl.

### Sequencing of the Chilean *Orientia* Isolates

Semi-nested PCR products from DNA preparations of four scrub typhus patients (Pt2–Pt5) were obtained using primers [16SO79F, 16SOR155F, and 16SOR1198R for *rrs*; and Otr47-263F, Otr47F, and Otr47-1404RL (GATTTACTTAT TAATGTTAGGTAAAGCAATGTAAAGCAT) for *htrA*] and amplification conditions described previously (7, 13). The amplicons of 986 bp of *rrs* and 1,421 bp of *htrA* were sequenced using Sanger method on a 3500 Genetic Analyzer (Thermo Fisher, Waltham, MA, United States) following procedures described previously (13). The sequences were submitted to GenBank with accession numbers: MZ773885 to MZ773888 for *rrs* from Pt2 to Pt5, respectively; and MZ773889 to MZ773891 for *htrA* from Pt2 to Pt4, respectively.

### Design of the Primers and the Probe for Orien16S Quantitative Real-Time PCR Assay

**FIGURE 1:**
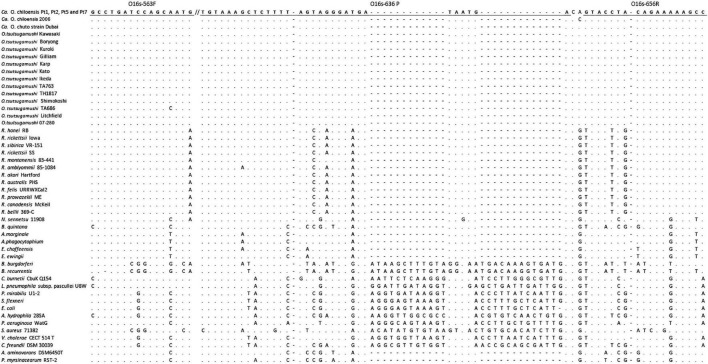
Alignment of the *rrs* sequence at primer and probe sites.

DNA sequences of *rrs* and *htrA* from various strains of *O. tsutsugamushi*, *Ca.* O. chuto, and *Ca*. O. chiloensis were studied to design the genus-specific qPCR assay; *rrs* was selected as the target gene due to the suitability of conserved sequences among all *Orientia* species assessed. The *rrs* sequences of 12 strains of *O. tsutsugamushi* from different geographical regions (Australia, New Guinea, Burma, Japan, Thailand, and South Korea), one strain of *Ca.* O. chuto (from United Arab Emirates) (6), and the initial isolate of *Orientia* species from Chile (3) were downloaded from GenBank. Sequences of the remaining four Chilean isolates used were obtained during this study. In addition, sequences of 12 *Rickettsia* species and nine other species of related Rickettsiales as well as 11 other bacteria were included. All sequences were aligned using the ClustalW within MEGA 7 (15). Then, the *rrs* conserved sequence fragments of *Orientia* species, which varied appreciably from other non-*Orientia* species, were used to design the primers and probe of the novel Orien16S qPCR assay (Figure 1). The suitability of the sequences as primers and probes were evaluated utilizing Beacon Designer software (Premier Biosoft, San Francisco, CA, United States). The selected primers (O16s-563F, 5′-GCCTGATCCAGCAATG-3′ and O16s-656R 5′-GGCTTTTTCTGTAGGTAC-3′) and the TaqMan probe (O16s-636 P 5′-FAM-TCATTATCAT CCCTACTAAAAGAGCTTTACA-BHQ-1-3′) were finalized after BLAST*™* search for specificity.

### Optimization of Orien16S TaqMan Quantitative Real-Time PCR Assay

The concentrations of the primers, the probe, and MgCl_2_ were optimized using Platinum Quantitative PCR SuperMix-UDG (Thermo Fisher, Waltham, MA, United States) and run on a StepOne Plus (Thermo Fisher) thermocycler system. Primer concentrations were varied simultaneously from 0.2 to 0.7 μM with steps of 0.1 μM, probe concentrations were varied from 0.1 to 0.5 μM (in steps of 0.1 μM), and the concentrations of MgCl_2_ were varied from 3 to 7 mM (in steps of 1 mM). The annealing temperature was optimized in the range from 56 to 61°C (in steps of 1°C).

The optimized conditions used for the final evaluation of Orien16S qPCR assay were: primers and probe at 0.3 μM, MgCl_2_ at 6 mM, and annealing/elongation temperature at 58°C; each 20 μl reaction for all the qPCR assays contained 2 μl of template DNA. The cycler parameters included: incubation at 50°C for 2 min (to allow for UDG contained within the master mix to function); initial denaturation at 95°C for 2 min; 45 cycles of denaturation at 95°C for 15 s; and annealing/elongation at 58°C for 30 s.

### Comparison of Orien16S Quantitative Real-Time PCR Assay With Otsu47 Quantitative Real-Time PCR Assay

The amplification results (Ct values) of Orien16S were compared to those of another *Orientia* qPCR assay, Otsu47, using identical *Orientia* DNA samples. Otsu47 is a qPCR assay developed for specifically detecting *O. tsutsugamushi* (12). This assay targets a portion of *htrA* and demonstrated high sensitivity and specificity detecting all *O. tsutsugamushi* strains tested in a previous study (12). The plasmid pOrien6, was used as positive control for both assays at 10^3^ copies/μl concentration.

### Application of Orien16S in Chile

After its development and validation in the Naval Medical Research Center, the novel Orien16S assay was implemented in the molecular laboratory of the Chilean Rickettsia and Zoonosis Research Group in Santiago, Chile, where it was adapted to the LightCycler 2.0 and LightCycler 480 platforms (Roche Life Science, Basel, Switzerland). The assay was applied within our ongoing surveillance project to detect *Orientia* DNA in suspected scrub typhus patients in Chile. The project was approved by the Comité Ético Científico, Pontificia Universidad Catolica de Chile in Santiago, Chile (#12–170 and #160816007) and the Naval Medical Research Center, Silver Spring, MD, United States (PJT-16–24). Orien16S was used to screen clinical samples and the positive samples were then confirmed by nested PCR assays using *Orientia*-specific targets, as previously described (7). All cases were acquired in southern Chile, except for one, which represented an imported scrub typhus case from South Korea (16). Further information on sample processing as well as clinical and epidemiological features of the cases can be found elsewhere (5, 16–18). Furthermore, the Orien16S assay was applied within a vector study on Chiloé Island, which collected and identified trombiculid mites. Mite pools were screened by Orien16S for the presence of *Orientia* DNA and subsequently confirmed by *Orientia*-specific nested PCR (11).

## Results

The sequences of the forward primer (16 bp), reverse primer (17 bp), and probe (31 bp) of the Orien16S qPCR were 100% identical to all species/stains of *Orientia* used in the sequence alignment except for *O. tsutsugamushi* strain TA686, which had 1 bp difference in the forward primer site (Figure 1). Primers were designed to produce a 94 bp PCR product, and the TaqMan probe was created to detect this product as a reverse complimentary sequence. Under the optimized conditions described above, a standard curve was generated using serial dilutions of pOrien6, ranging from 10^8^ to 0.1 copies/reaction (*n* = 13 points), which showed a *R*^2^ value of 0.996 and an assay performance efficiency of 100.026%. The LOD of Orien16S was assessed using pOrien6 at 1, 3.16, 5, and 10 copies/reaction. Since samples containing 10 copies/reaction were consistently positive (100% of 30 runs), 10 copies was determined as the LOD.

The validation of Orien16S utilized a panel of 85 DNA preparations, including a variety of rickettsial microorganisms (Table 1). The new assay correctly identified all 47 *Orientia* specimens, which included *O. tsutsugamushi* and the two newly described *Candidatus* species *Ca.* O. chiloensis and *Ca.* O. chuto, thus demonstrating a genus-specific sensitivity of 100%. The determination of specificity utilized a panel of 38 samples, consisting of 18 *Rickettsia* species, 4 species of Anaplasmataceae, and 11 other bacterial species, as well as human and mouse DNA (Table 1). No false positive reactions were observed in this panel.

The head-to-head comparison of Orien16S with a previously established qPCR assay for *O. tsutsugamushi* (Otsu47) demonstrated that both detected 41 *O. tsutsugamushi* strains with similar average replication cycle thresholds (Cts) of 27.9 and 28.7, respectively (range 18.8–36.3 and 20.5–37.6, respectively; Table 2). However, Orien16S assay detected *Ca.* O. chuto more efficiently (Ct 28.4 vs Ct 42.8) and identified all five *Ca.* O. chiloensis samples, which Otsu47 failed to detect (Table 2).

**TABLE 2:** Comparison of the amplification characteristics (Ct values) of Orien16S and Otsu47 qPCR assays with samples of *Orientia tsutsugamushi*, *Ca.* Orientia chuto, and *Ca.* Orientia chiloensis.

	qPCR Ct^a^ values			qPCR Ct values	
			
Strain	Orien16S	Otsu47	Strain	Orien16S	Otsu47
*O. tsutsugamushi* Karp	28.29	30.19	*O. tsutsugamushi* TA686	28.08	28.25
*O. tsutsugamushi* Kato	29.32	30.24	*O. tsutsugamushi* Volner	26.23	27.36
*O. tsutsugamushi* Gilliam	27.99	29.11	*O. tsutsugamushi* Ikeda	21.66	22.19
*O. tsutsugamushi* TA-763	21.73	22.77	*O. tsutsugamushi* Domrow	28.89	30.82
*O. tsutsugamushi* TH-1814	29.46	30.27	*O. tsutsugamushi* Middleton	30.75	32.03
*O. tsutsugamushi* TH-1817	29.03	30.26	*O. tsutsugamushi* Hicks	30.5	31.3
*O. tsutsugamushi* AFC-3	20.59	22.19	*O. tsutsugamushi* Faulkner	21.89	20.68
*O. tsutsugamushi* AFC-30	31.58	32.65	*O. tsutsugamushi* UT76	29.78	30.73
*O. tsutsugamushi* AFPL-12	30.07	31.26	*O. tsutsugamushi* UT221	30.82	32.05
*O. tsutsugamushi* MAK-110	28.68	29.18	*O. tsutsugamushi* FPW1038	30.45	31.24
*O. tsutsugamushi* MAK-119	28.87	29.98	*O. tsutsugamushi* FPW2016	27.49	28.17
*O. tsutsugamushi* MAK-243	28.57	29.36	*O. tsutsugamushi* CRF136	33.58	33.44
*O. tsutsugamushi* 18030642	26.65	26.97	*O. tsutsugamushi* TM1073	36.26	37.63
*O. tsutsugamushi* 18-032460	18.78	20.7	*O. tsutsugamushi* TM1324	34.69	35.86
*O. tsutsugamushi* BSR178	20.85	20.75	*O. tsutsugamushi* UT661	36.12	35.38
*O. tsutsugamushi* Buie	29.22	29.8	*O. tsutsugamushi* Sido	22.84	23.3
*O. tsutsugamushi* Calcutta	28.31	29.88	*O. tsutsugamushi* Kawasaki	29.23	29.42
*O. tsutsugamushi* Brown	29.21	30.29	*Ca*. O. chuto	28.43	42.82
*O. tsutsugamushi* Citrano	24.32	25.35	*Ca.* O. chiloensis Pt1	42.3	Negative
*O. tsutsugamushi* Garton	29.45	30.46	*Ca.* O. chiloensis Pt2	36.56	Negative
*O. tsutsugamushi* Woods	30.32	30.91	*Ca.* O. chiloensis Pt3	31.25	Negative
*O. tsutsugamushi* 18-032111	19.43	20.52	*Ca.* O. chiloensis Pt4	26.27	Negative
*O. tsutsugamushi* TH1812	27.53	28.61	*Ca*. O. chiloensis Pt5	32.25	Negative
*O. tsutsugamushi* TA678	25.77	26.87	pOrien6^b^ 1000 copies	28.67	28.68

*^a^Ct, cycle threshold. The cut-off Ct values for Orien16S and Otsu47 were not applied since reliable and consistent exponential curves were presented in all samples with a Ct value.*

*^b^pOrien6 plasmid served as positive control.*

After its development, the new qPCR assay, Orien16S, was implemented in the molecular laboratory of the Chilean Rickettsia and Zoonosis Research Group, Santiago, Chile, and applied within our ongoing clinical surveillance and vector studies. In accordance with the validation data, Orien16S proved to be a reliable tool for the diagnosis of scrub typhus acquired in Chile and one imported *O. tsutsugamushi* case (4, 5, 7, 16–18). The new assay correctly identified 23 eschar samples and 5 buffy coat preparations, with Ct values ranging from 24.65 to 35.54 (Table 3, assay cut-off Ct = 36), all Orien16S positive samples were confirmed by *Orientia*-specific nested PCR protocols (7). The new assay was also used within a field project to investigate *Orientia* infection in chigger mites collected from captured rodents on Chiloé Island (11). As shown in Table 3, four mite pools confirmed by *Orientia*-specific nested PCR (targeting *rrs*) were identified by Orien16S with Ct values ranging from 30.94 to 33.48.

**TABLE 3:** Amplification results (Ct values) of new Orien16S qPCR assay in specimens from human scrub typhus cases and mite pools from Chiloé Island.

Clinical samples				

No.	Patient sex/age (years)	Sample type	Orien16S Ct^a^ value	References
1	Male/43	Eschar	31.76	(5, 7)
2	Male/56	Eschar	24.65	(5, 7)
3	Male/56	Buffy coat	35.62	(5, 7)
4	Male/25	Eschar	29.76	(5, 18)
5	Male/69	Eschar	25.65	(5, 7)
6	Male/69	Buffy coat	29.77	(5, 7)
7	Female/22	Eschar	27.38	(5, 7)
8	Male/25	Eschar	28.09	(5, 7)
9	Male/39	Eschar	28.76	(5, 7)
10	Male/39	Buffy coat	26.8	(5, 7)
11	Male/28	Eschar	31.58	(5, 7)
12	Male/28	Buffy coat	32.85	(5, 7)
13	Female/21	Eschar	25.99	(5, 7)
14	Male/20	Eschar	29.41	(17)
15	Male/17	Eschar	35.54	(17)
16	Male/55	Eschar	34.16	(4, 7)
17	Male/73	Eschar	30.97	(7)
18	Female/49	Eschar	27.12	(7)
19	Female/49	Buffy coat	35.12	(7)
20	Female/30	Eschar	24.35	(7)
21	Male/54	Eschar	25.12	(7)
22	Male/63	Eschar	26.28	(7)
23	Female/23	Eschar	28.4	(7)
24	Male/53	Eschar	23.45	(7)
25	Male/41	Eschar	24.55	(7)
26	Female/54	Eschar	26.82	(7)
27	Male/44	Eschar	29.39	(18)
28^b^	Male/62	Eschar	29.23	(16)

**Mite samples**				

**No.**	**Sample ID**	**Sample type**	**Orien16S Ct value**	**References**

1	A olivacea/site4_1	Mite pool	33.48	(11)
2	A olivacea/site4_4	Mite pool	30.94	(11)
3	A sanborni/site4_1	Mite pool	33.14	(11)
4	A olivacea/site6_1	Mite pool	31.64	(11)

*^a^Ct, cycle threshold. Ct value of 36 was used as the cut-off for Orien16S.*

*^b^The infection for sample No. 28 was from South Korea and the rest were acquired in Chile.*

## Discussion

Similar to other rickettsial diseases, scrub typhus can be a severe and potentially life-threatening infection and therefore requires rapid and effective treatment. However, The generalized flu-like symptoms are clinically indistinguishable from many febrile illnesses, such as dengue fever and other rickettsioses; the characteristic eschar for scrub typhus can also be caused by other bacterial infections (19); in addition, the strictly intracellular nature of these causative pathogens and the requirement of BSL3 labs make the isolation and culture impossible in most areas of the world. Due to these facts, timely and reliable identification is challenging. Molecular methods are nowadays considered the indispensable tool and are becoming a standard for the detection and identification of this group of microorganisms (20).

The Naval Medical Research Center has developed a qPCR assay, Otsu47, to detect *O. tsutsugamushi* (12). This assay has been used successfully around the world both in clinical and vaccine studies within the tsutsugamushi triangle (21, 22). However, Otsu47 performed poorly with the detection of *Ca.* O. chuto (13), and it did not recognize *Orientia* DNA from the first reported scrub typhus patient in Chile (3). Subsequent cases from Chile confirmed that Otsu47 and other established *O. tsutsugamushi-specific* PCR assays failed to consistently identify *Orientia* samples from this region (authors’ unpublished observations). The unsatisfactory performance of these detection methods targeting *O. tsutsugamushi* became comprehensible, when the cause of scrub typhus in Chile was identified as a novel *Orientia* agent distinct from *O. tsutsugamushi* and *Ca.* O. chuto (7).

In response to this diagnostic gap, we designed and validated a novel qPCR assay, Orien16S, which is based on genus-specific *rrs* sequences including those of *Ca*. O. chiloensis. After optimization, the LOD of this assay was determined to be 10 copies/reaction. Using a broad panel of *O. tsutsugamushi* samples of different geographical origins as well as samples of the two newly designated *Orientia* species (*Ca*. O. chuto and *Ca*. O. chiloensis), Orien16S demonstrated an excellent analytical sensitivity (100%). The assay also showed a high level of genus-specificity among a broad control panel of 22 species within the Rickettsiaceae and Anaplasmataceae families.

After its implementation in Chile, the Orien16S successfully detected *Orientia* DNA extracted from clinical samples of new or previously confirmed scrub typhus cases, including serum from the first Chilean case from 2006 (3) and a variety of sample types (blood, eschar material, and eschar swabs) from all other confirmed Chilean patients as well as the first known imported scrub typhus case in Chile caused by *O. tsutsugamushi* (Table 3). Due to its reliability and rapid turnaround time, it became the primary diagnostic tool within our ongoing scrub typhus surveillance in Chile. In the future, this new molecular tool may help to detect cases in other parts of Latin America, where scrub typhus caused by *Ca.* O. chiloensis might be present (23). Given that the Orien16S assay also detects *Ca.* O. chuto, it should be useful in the Middle East and Africa, where this species and/or related species may be endemic (2). Further evaluation of Orien16S by other research groups is needed to conclusively prove its utility for identifying orientiae in clinical samples.

In addition to clinical diagnosis, Orien16S was applied to identify *Orientia* DNA in chigger mites, collected in endemic areas in southern Chile (11); the assay reliably identified four *Orientia*-infected mite pools and the positive results were confirmed by nested PCR. The observed Ct values of those samples were within the same range as in clinical samples. However, we observed some pooled mite samples that produced higher Ct values by Orien16S but were not amplified by nested-PCR assays (data not shown). Some of those samples would be interpreted as positive, if their Ct values were within the range of the LOD (Ct < 36) and repeatedly demonstrating a consistent melting curve. Ct values well above 35 have been treated as positive as reported in a study on *O. tsutsugamushi* in chigger mites in Asia (22). Since trombiculid mites are less studied than other arthropod vectors, methodological standards for sample preparation and DNA extraction are lacking (10), higher Ct values from those mites might be due to methods used were not optimal. Interestingly, all *Orientia*-infected mites from our field study belonged to a newly described trombiculid species, *Herpetacarus eloisae* (24). Orien16S was also successfully applied to demonstrate the presence of *Orientia* DNA in rodent tissues from Chiloé Island (authors’ unpublished observations).

## Conclusion

In conclusion, the new qPCR assay Orien16S is a highly sensitive and specific tool for the genus-specific detection of *Orientia* DNA. In our clinical and epidemiological studies in Chile, it has proven its usefulness to identify *Ca*. O. chiloensis in different types of patient samples and in trombiculid mites. The ability of the assay to detect all known *Orientia* species suggests that it might help to clarify the possible existence of scrub typhus in regions outside the tsutsugamushi triangle including Latin America, the Middle East, and Africa.

## Data Availability Statement

The datasets presented in this study can be found in online repositories. The names of the repository/repositories and accession number(s) can be found in the article/supplementary material.

## Ethics Statement

The studies involving human participants were reviewed and approved by the Comité Ético Científico, Pontificia Universidad Catolica de Chile in Santiago, Chile (#12–170 and #160816007) and the Naval Medical Research Center, Silver Spring, MD, United States (PJT-16–24). Written informed consent to participate in this study was provided by the participants or their legal guardian/next of kin.

## Author Contributions

AR and JJ: conceptualization. JJ: methodology and visualization. JJ, CM-V, and GA-J: validation. JJ and TW: formal analysis and writing—original draft preparation. JJ, KA, and GA-J: investigation. AR and CF: resources. JJ and CM-V: data curation. AR, CF, CM-V, KA, and GA-J: writing—review and editing. AR, CF, and TW: supervision. CF and KA: project administration and funding acquisition. All authors have read and agreed to the final version of the manuscript.

## Author Disclaimer

The views expressed in this article are those of the author and do not necessarily reflect the official policy or position of the Department of the Navy, Department of Defense, the U.S. Government or the Henry M. Jackson Foundation for the Advancement of Military Medicine, Inc. CF is an employee of the U.S. Government and this work was prepared as part of her official duties. Title 17 U.S.C. §105 provides that copyright protection under this title is not available for any work of the United States Government. Title 17 U.S.C. §101 defines a U.S. Government work as a work prepared by a military service member or employee of the U.S. Government as part of that person’s official duties.

## Conflict of Interest

JJ and AR are employed by the Henry M. Jackson Foundation for the Advancement of Military Medicine, Inc. The remaining authors declare that the research was conducted in the absence of any commercial or financial relationships that could be construed as a potential conflict of interest.

## Publisher’s Note

All claims expressed in this article are solely those of the authors and do not necessarily represent those of their affiliated organizations, or those of the publisher, the editors and the reviewers. Any product that may be evaluated in this article, or claim that may be made by its manufacturer, is not guaranteed or endorsed by the publisher.

## Footnotes

1
https://blast.ncbi.nlm.nih.gov/
